# Maternal Rumen-Protected Leucine Supplementation Enhances Placental Nutrient Transport Capacity and Increases Birth Weight in Hu Sheep

**DOI:** 10.3390/vetsci13060592

**Published:** 2026-06-18

**Authors:** Qin Gao, Chong Yuan, Shanglai Li, Hua Yang, Zongyou Wei, Yanli Zhang

**Affiliations:** 1 Jiangsu Livestock Embryo Engineering Laboratory, Nanjing Agricultural University, Nanjing 210095, China; 2022205022@stu.njau.edu.cn (Q.G.); 2024105044@stu.njau.edu.cn (C.Y.); 2021105035@stu.njau.edu.cn (S.L.); yanghua004@njau.edu.cn (H.Y.); 2 Taicang Animal Husbandry and Veterinary Station, Jiangsu Science and Technology Backyard, Enterprise Graduate Workstation, Taicang 215400, China; tcnltz@163.com

**Keywords:** perinatal ewe, rumen-protected leucine, growth performance, placenta

## Abstract

Leucine, an essential branched-chain amino acid, serves not only as a substrate for protein synthesis but also as a key regulator of placental function and fetal development. This study investigated the effects of dietary supplementation with RP-Leu during late gestation on placental development and offspring performance in Hu sheep. Sixty twin-pregnant ewes at day 80 of pregnancy were randomly assigned to either a control group (fed a basal diet) or an RP-Leu group (fed a basal diet supplemented with 19 g/day RP-Leu). The feeding trial lasted for 60 d. The ewes were slaughtered at day 140 of gestation. Maternal slaughter traits and fetal organ weights were recorded. Blood and milk samples were collected for milk composition analysis and targeted metabolomic profiling. Leucine supplementation significantly increased the percentage of milk fat content, total solid content, and the birth weight of lambs (*p* < 0.05). Improvements in placental morphology and antioxidant capacity were observed, including a significant increase in cotyledon density and a significant enhancement of catalase (CAT) activity (*p* < 0.05). Gene expression analysis indicated that the *NOS3*, *SLC38A1* and *FABP4* genes in the placental cotyledons (*p* < 0.05), and the *VEGFA, NOS3*, *SLC27A1* and *FABP4* genes were significantly upregulated in the maternal caruncles (*p* < 0.05). Plasma metabolomic profiling revealed increased L-glutamic acid levels and alterations in several amino acids, with pathway enrichment indicating involvement in amino acid metabolism and membrane transport processes. Transcriptomic analysis identified 739 differentially expressed genes, which were mainly enriched in the PI3K/Akt signaling pathway, ECM–receptor interaction pathway, and cytokine–cytokine receptor interaction pathway. Collectively, these findings suggest that RP-Leu supplementation during late gestation may enhance offspring growth by modulating amino acid metabolism, promoting placental development, and improving placental nutrient transport capacity, thereby supporting fetal growth and development.

## Simple Summary

This study examined the effects of supplementing the diets of pregnant sheep with rumen-protected leucine (RP-Leu), an essential amino acid, during late gestation and the early postpartum period. The aim was to evaluate potential benefits for both ewes and their lambs. Results showed that RP-Leu supplementation increased milk fat percentage and improved lamb birth weight. These positive effects may be associated with enhanced amino acid metabolism, improved placental development, and greater nutrient transfer to the fetus. Overall, dietary RP-Leu supplementation may serve as a promising nutritional strategy to improve reproductive performance in sheep.

## 1. Introduction

Branched-chain amino acids (BCAAs), including leucine (Leu), isoleucine (Ile), and valine (Val), are a group of essential amino acids that animals cannot synthesize on their own and must obtain from their diet [1]. In recent years, studies have shown that BCAAs not only serve as essential substrates for protein synthesis but also act as key signaling molecules involved in metabolic regulation, cell proliferation, and gene expression [2,3]. Among them, leucine plays a central role in nutrient sensing and metabolic regulation, influencing physiological processes such as energy metabolism, lipolysis, and insulin secretion [4,5,6,7]. In ruminants, BCAAs and their metabolites have been confirmed to participate in multiple physiological processes, including gastrointestinal digestion and absorption, tissue metabolism, mammary gland development, and immune system function. A better understanding of the regulatory roles of BCAAs, particularly leucine, may provide new opportunities for optimizing nutritional strategies and improving production efficiency in ruminants [8].

In recent years, the role of Leu in early-life developmental programming has attracted growing attention, particularly regarding its involvement in regulating placental function at the maternal–fetal interface [9,10]. The placenta is the sole organ responsible for the transfer of oxygen and nutrients from the mother to the fetus, and its development and functional capacity are critical determinants of fetal growth and long-term offspring health [11,12]. Impaired placental development or dysfunction is closely associated with fetal growth restriction, low birth weight, and reduced postnatal performance [13,14,15,16]. Consequently, nutritional interventions that promote placental development and enhance nutrient transport efficiency have become an important focus for improving reproductive performance in livestock production.

In ruminants, however, dietary leucine is extensively degraded by rumen microorganisms, which limits its bioavailability and constrains its application as a functional nutrient [17]. Rumen-protected leucine (RP-Leu) offers a practical approach to overcome this limitation by enabling leucine to escape ruminal degradation and reach the small intestine for absorption. RP-Leu provides a potential solution by allowing leucine to bypass ruminal degradation and reach the small intestine for absorption. In recent years, rumen-protected amino acids have been widely used in ruminant production. Studies have shown that the addition of rumen-protected methionine can improve intestinal barrier function, enhance reproductive performance, and promote metabolic health during the perinatal period [18]. The combined addition of rumen-protected lysine and methionine can enhance the growth performance of ruminants, promote the digestion and absorption of feed nutrients, and improve the quality of animal products. Despite these advances, little is known about the effects of RP-Leu supplementation during late gestation on placental development, nutrient transport function, and fetal growth in ruminants. Moreover, the molecular mechanisms underlying these responses remain largely unclear.

Increasing evidence suggests that amino acids regulate placental development and nutrient transport through multiple signaling pathways [19]. Among these, the PI3K/Akt signaling pathway plays a pivotal role in regulating placental growth, cellular metabolism, and the expression of nutrient transporters. Studies have demonstrated that rumen-protected methionine promotes fetal growth by upregulating placental PI3K/Akt signaling and amino acid transporters, including *SLC1A5*, *SLC7A5*, and *SLC38A6* [20]. In addition, other functional amino acids, such as arginine and glutamine, have also shown beneficial effects during pregnancy. For example, glutamine supplementation during late gestation has been reported to improve the growth performance of twin lambs [21]. Whether leucine exerts similar regulatory effects on placental nutrient transport and fetal development in ruminants has not yet been systematically investigated.

Therefore, we hypothesized that maternal RP-Leu supplementation during late gestation would enhance placental development and nutrient transport capacity by modulating amino acid metabolism, antioxidant status, and nutrient transporter expression, thereby promoting fetal growth. Accordingly, the objective of this study was to investigate the effects of RP-Leu supplementation on maternal metabolism, placental function, and offspring performance in Hu sheep and to explore the molecular mechanisms underlying these responses.

## 2. Materials and Methods

### 2.1. Management of Animals and Experimental Design

This experiment was conducted using Hu sheep at Dongling Ecological Livestock Breeding Professional Cooperative, Taicang City, China. Following a 10 d adaptation period, the experiment was conducted from day 80 of gestation until 30 d postpartum. Ewes aged 2–3 years and weighing 52.69 ± 1.75 kg were selected according to pedigree records and naturally mated. At day 80 of gestation, ewes carrying twin fetuses were identified by ultrasonography and housed individually. Sheep pens and feeding equipment were thoroughly cleaned and disinfected. Using a completely randomized design, 60 twin-pregnant ewes were randomly assigned to one of two treatments (*n* = 30 per treatment) [22,23]: (1) a control group (Con), fed the basal diet; or (2) an RP-Leu group, fed the basal diet supplemented with 19 g/d RP-Leu.

The experimental diet consisted of concentrate and roughage mixed at a ratio of 40:60 on a dry matter basis. The ingredient composition and nutrient levels of the basal diet are presented in Table 1. RP-Leu was thoroughly mixed with the basal diet and offered once daily during the morning feeding. Ewes were fed twice daily and had free access to fresh water throughout the experiment.

The RP-Leu supplementation level (19 g/d) was determined based on previous studies [24] and a preliminary trial, in which this dose improved lamb birth weight without adverse effects on feed intake, behavior, or health status. Based on the nutrient requirements of pregnant ewes and the results of the preliminary trial, each ewe received 3.0 kg/d of the basal diet (as-fed basis). Amount of feed offered and refusals were recorded daily. Representative feed samples were collected weekly for dry matter determination. The basal diet contained 59.90% dry matter, resulting in an average dry matter intake of approximately 1.80 kg/d per ewe. Since the actual wet weight intakes of each group were the same, there was no difference in the calculated DMI among the treatment groups.

At approximately day 140 of gestation, eight ewes per treatment were randomly selected for tissue collection. Following a 24 h feed withdrawal and 12 h water deprivation period, ewes were electrically stunned and immediately exsanguinated via the carotid artery. Maternal, placental, and fetal tissue samples were collected for subsequent analyses. The remaining ewes were maintained under the same feeding regimen until parturition and throughout the 30 d postpartum period. The survival rate of lambs during the first 7 d after birth was 100%.

This study was approved by the Animal Welfare and Ethics Committee of Nanjing Agricultural University (Approval No. SYXK (Su) 2022-0031). All procedures involving animals were conducted in accordance with the Guide for Ethical Review of Laboratory Animal Welfare (GB/T 35892-2018 [25]) and relevant national regulations.

### 2.2. Measurement Indicators and Methods

#### 2.2.1. Determination of RP-Leu Transrumen Rate

RP-Leu was supplied by Shanghai Yimengs Chemical Co., Ltd. (Shanghai, China) and coated by Hangzhou Kangdequan Feed Co., Ltd. (Hangzhou, China). The coating materials primarily consisted of hydrogenated palm oil, silicon, and sodium carboxymethyl cellulose. The rumen bypass rate of RP-Leu was determined using fistulated sheep provided by Jiangsu Academy of Agricultural Sciences (Nanjing, China). All animals used for the bypass evaluation were managed under feeding and husbandry conditions similar to those of the experimental ewes.

The determination procedure followed the Inner Mongolia Autonomous Region standard “Stability Test of Rumen-Protected Amino Acids” (DB15/T 2154–2021 [26]). Residual leucine in nylon bags after ruminal incubation was quantified according to GB 5009.124 [27], and the rumen bypass rate was subsequently calculated based on the residual amino acid concentration.

The rumen protection rate (RPR, %) was calculated as follows:

RPR (%) = (A_1_/A_0_) × 100%

where A_0_ is the initial amount of the amino acid in the sample, and A_1_ is the amount remaining after in situ/in vitro ruminal digestion.

#### 2.2.2. Determination of Feed Composition

Feed samples were collected at 07:00 and 14:00 h during weeks 3, 6, and 8 of the experimental period. At each sampling time, feed samples were thoroughly mixed and homogenized using the coning and quartering method to obtain representative subsamples. Approximately 500 g of each subsample was sealed and stored at −20 °Cuntil analysis.

Prior to chemical analysis, feed samples were dried in a forced-air oven at 65 °C to constant weight, ground through a 40-mesh screen using a laboratory mill, and stored in sealed containers. Dry matter (DM) content was determined according to the method described by Zhang et al. [28]. The concentrations of gross energy (GE), crude protein (CP), ether extract (EE), neutral detergent fiber (NDF), acid detergent fiber (ADF), calcium (Ca), and phosphorus (P) were analyzed using standard procedures.

#### 2.2.3. Production Performance Measurement

Referring to the standards recorded in the breeding sheep archives of NY/T 1236-2023 [29], the traditional body size measurement method is used to measure the body length, body height, chest circumference, body length, and tube circumference of lambs with a soft ruler. When using the measuring tool, pay attention to the appropriate tightness, gently align the measuring point, and do not hang or squeeze the skin to measure.

#### 2.2.4. Calculation of Placental Index

Placental measurements were performed in a blinded manner with respect to treatment groups to minimize observer bias.

Cotyledon number and diameter: The fetal membranes surrounding each cotyledon were carefully excised along the cotyledonary margin using surgical scissors. The total number of cotyledons per placenta was recorded. Twenty intact cotyledons without visible damage were randomly selected from each placenta, and their diameters were measured using a vernier caliper. The mean cotyledon diameter was then calculated for each placenta.

Placental mass: After parturition, the placenta was carefully separated from the uterus and fetal membranes, rinsed with Dulbecco’s phosphate-buffered saline (DPBS; Gibco) to remove residual blood and debris, gently blotted dry with absorbent paper, and weighed using an electronic balance.

Total cotyledon area: The area of each selected cotyledon was estimated based on its measured diameter, assuming a circular shape. Total cotyledon area was calculated as the product of the mean cotyledon area and the total number of cotyledons.

Placental efficiency: Placental efficiency was calculated as the ratio of total litter birth weight (kg) to placental mass (kg).

Cotyledon density: Cotyledon density was calculated as the number of cotyledons per unit placental mass (cotyledons/kg placenta).

Cotyledonary carrying efficiency: Cotyledonary carrying efficiency was calculated as the ratio of total litter birth weight (kg) to total cotyledon area (cm^2^).

#### 2.2.5. Determination of Milk Quality Index

On day 30 of the lactation period of ewes, milk samples were collected within 3 h after the morning feeding. Prior to sampling, ewes were separated from their lambs for 2 h to allow milk accumulation. Approximately 50 mL of milk was manually collected from each ewe and thoroughly mixed to obtain a representative sample. The milk samples were then transported to the Jiangsu Dairy Cow Production Performance Testing Center for analysis.

Milk composition was determined using a milk analyzer (FOSS MilkoScan FT+ 200, FOSS, Hillerød, Denmark). The analyzed parameters included lactose, milk fat, milk protein, solids-not-fat (SNF), milk urea nitrogen (MUN), and somatic cell count (SCC).

#### 2.2.6. Measurement of Blood Indicators

On days 30, 60 and 90 of the experiment, jugular blood was collected into vacuum procoagulant tubes before morning feeding. The samples were allowed to clot at room temperature for 30 min and then centrifuged at 3,500 rpm for 10 min to obtain serum. The serum was carefully separated, transferred into 1.5 mL centrifuge tubes, and stored at −80 °C until further analysis.

Serum biochemical parameters were analyzed using a fully automatic biochemical analyzer (AU 5800, Olympus, Tokyo, Japan). Serum antioxidant indices, including catalase (CAT; A007-1-1), total antioxidant capacity (T-AOC; A015-2-1), total superoxide dismutase (T-SOD; A001-3-2), glutathione peroxidase (GSH-Px; A005-1-2), and malondialdehyde (MDA; A003-1-2), were determined according to the manufacturer’s instructions using commercial assay kits (Nanjing Jiancheng Bioengineering Institute, Nanjing, China). The assay principles were based on the ammonium molybdate method (CAT), ABTS method (T-AOC), WST-1 method (T-SOD), colorimetric method (GSH-Px), and thiobarbituric acid (TBA) method (MDA), respectively.

Serum leucine metabolite-related indicators, including α-ketoisocaproate (KIC; ANS-E51100S) and β-hydroxy-β-methylbutyrate (HMB; ANS-E51135S), were measured according to the manufacturer’s protocols (AngleGene, Nanjing, China).

#### 2.2.7. Real-Time Quantitative Polymerase Chain Reaction

Total RNA was extracted and reverse-transcribed into complementary DNA (cDNA) using the PrimeScript RT reagent Kit with gDNA Eraser (TaKaRa Biotechnol ogy, Dalian, China), according to the manufacturer’s instructions.

Gene-specific primers were designed using the NCBI Primer-BLAST online tool (Primer3 version 2.5.0) and synthesized by Qingke Biotechnology Co., Ltd., Nanjing, China. The specificity and amplification efficiency of all primers were verified by conventional PCR followed by agarose gel electrophoresis.

Quantitative real-time PCR (qRT-PCR) was performed using a QuantStudio™ 5 Real-Time PCR System (Applied Biosystems, Woburn, MA, USA) with SYBR Green chemistry, following the manufacturer’s protocol. The qPCR reaction procedure is as follows: pre-denaturation at 95 °C for 30 s; cycle 40 times at 95 °C for 10 s, 60 °C for 30 s, 95 °C for 15 s, 60 °C for 60 s, and 95 °C for 15 s. After amplification was completed, quantification was carried out by the 2^−ΔΔCt^ method, with Glyceraldehyde 3-phosphate dehydrogenase (GAPDH) gene serving as an internal reference. Each trial was independently repeated three times. The specific primer sequences for qPCR are shown in Table 2.

#### 2.2.8. Western Blot Analysis

Total tissue proteins were extracted using RIPA lysis buffer (ThermoFisher Scientific, 78501, Waltham, MA, USA) containing protease inhibitors and phosphatase inhibitors. After lysis on ice for 30 min, the samples were centrifuged at 12,000 r/min for 15 min at 4 °C, and the supernatant was collected as the total protein sample. The protein concentration was detected using a BCA protein quantification kit (Beyotime, P0012, Shanghai, China). According to the quantification results, the protein loading amount of each sample was adjusted. After adding 5 × SDS loading buffer, the samples were boiled in a boiling water bath for 10 min to fully denature the proteins. An equal amount of denatured protein samples was subjected to SDS-polyacrylamide gel electrophoresis (SDS-PAGE) (Biosharp, BL502B, Beijing, China) for separation. After electrophoresis, the proteins were transferred to a PVDF membrane. After the transfer was completed, the membrane was blocked with 5% non-fat milk at room temperature for 1.5 h to reduce non-specific binding. After blocking, the membrane was incubated with the primary antibody dilution (Biosharp, BL1027B, Beijing, China) on a shaker at 4 °C overnight. The next day, the membrane was washed 3 times with TBST buffer for 10 min each time, and then the corresponding secondary antibody dilution (Biosharp, BL5678A, Beijing, China) was added and incubated on a shaker at room temperature for 1 h. After washing the membrane 3 times with TBST again, the membrane was developed using ECL chemiluminescence developing solution (Biosharp, BL520B-2, Beijing, China) in a darkroom, and the band images were collected through a gel imaging system. Using α-Tubblin as an internal reference, the gray value of the target protein band was quantitatively analyzed using ImageJ (Version 1.54p) software, and the relative expression level of the protein was represented by the ratio of the gray value of the target protein to that of the internal reference protein.The relevant information of the antibodies used is shown in Table 3.

### 2.3. RNA Sequencing (RNA-Seq) and Data Processing

Total RNA was isolated with TRIzol, quantified by Nanodrop and Agilent (RQN > 6.5; ≥1 µg, ≥30 ng µL^−1^, 1.8 ≤ 260/280 ≤ 2.2). Poly-A mRNA was fragmented, reverse-transcribed, end-repaired, adapter-ligated, PCR-amplified and sequenced on NovaSeq X Plus. Clean reads (fastp) were aligned (HISAT2) and quantified (RSEM). Differentially expressed genes (DEGs) were identified using DESeq2, applying thresholds of |fold change| ≥ 2 and adjusted *p*-value (padj) < 0.05. Functional annotation of DEGs was performed through GO and KEGG enrichment analyses with a significance threshold of padj < 0.05.

### 2.4. Plasma Targeted Amino Acid Metabolomics Analysis

A total of 50 μL of plasma samples collected from ewes at 140 days of gestation were used for targeted amino acid metabolomics analysis. Water, 0.15% sodium deoxycholate, a mixed internal standard solution (Lys-d4, Trp-d5, Gln-d4), and trichloroacetic acid were sequentially added for protein precipitation and sample preparation.

After ultrasonication, low-temperature precipitation, and centrifugation, the supernatant was collected, diluted, and filtered to obtain the final analytical solution.

Metabolite detection was performed using an LC–ESI–MS/MS system (QTRAP 6500+, AB Sciex, Framingham, MA, USA). Raw data were automatically processed for peak identification and integration using AB Sciex OS software(Version 3.4), followed by manual inspection to ensure data quality. Metabolite concentrations were calculated based on external standard curves, and the resulting data were uploaded to the Shanghai Meiji Biological Cloud Platform for further analysis.

Multivariate statistical analyses, including principal component analysis (PCA) and orthogonal partial least squares discriminant analysis (OPLS-DA), were performed using the ropls package in R(Version 3.3.1) software. Model robustness was validated using 7-fold cross-validation. Differential metabolites were identified using Student’s *t*-test (*p* < 0.05). Metabolic pathway annotation was performed using the Kyoto Encyclopedia of Genes and Genomes (KEGG) database, and pathway enrichment analysis was conducted using Fisher’s exact test.

### 2.5. Data Processing

Statistical analyses were performed using SPSS (Version 23.0, IBM Corporation, Armonk, NY, USA). For statistical data (including growth traits, repeated-measures indicators, and gene expression data), the Shapiro–Wilk test was first used to assess normality, and the Levene test was used to evaluate the homogeneity of variance. Variables that conformed to the normal distribution and had homogeneous variance were analyzed using the independent-samples *t*-test, and the results were presented as mean ± SEM. Data that did not meet the assumptions of normality or homogeneity of variance were analyzed using the Mann–Whitney U test. When analyzing lamb-level traits (such as birth weight and growth performance), to correct for the non-independence of data from twin lambs born to the same ewe, “ewe” was included as a random effect in the linear mixed model. For multiple-comparison analyses such as RNA-seq, the false-discovery rate (FDR) method was used for multiple testing correction to control the false-positive rate. All analyses provided exact *p*-values (e.g., *p* = 0.023), and the significance level was set at *p* < 0.05. Data were initially summarized in Microsoft Excel (Version 2016), and graphs were generated using Origin (Version 2025b, Origin Lab Corporation, Northampton, MA, USA). In tables and charts, *p* < 0.05 indicates a significant difference, *p* < 0.01 indicates an extremely significant difference, and *p* > 0.05 indicates no significant difference.

## 3. Results

### 3.1. Determination of RP-Leu Ruminal Degradability

The in situ ruminal degradation characteristics of rumen-protected leucine (RP-Leu) were evaluated using the nylon bag technique. The specific values are shown in Table 4. The 12 h ruminal degradation rate of RP-Leu was 54.87%, while the ruminal bypass (escape) rate was approximately 90%. Based on these degradation characteristics, RP-Leu was supplemented at 19 g/day, corresponding to an estimated effective leucine supply of approximately 10 g/day.

### 3.2. Effects of Perinatal Ewe Supplementation of RP-Leu on the Production Performance of Ewes

**Figure 1:**
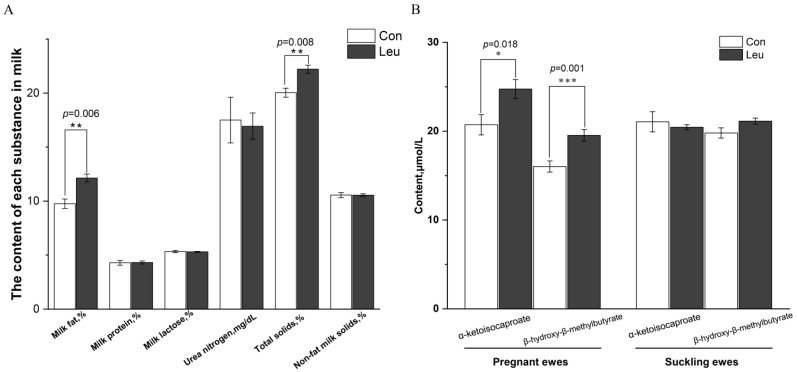
Effects of RP-Leu supplementation on milk quality (**A**) and blood leucine metabolites (**B**) of perinatal ewes. Note: Con = The control group; Leu = The leucine group. The data in (**A**) are from postpartum ewes, with 8 ewes in each group; the data in (**B**) are from slaughtered ewes, with 8 ewes in each group. Asterisks (*) above the error bar indicate significant differences between the two groups (* *p* < 0.05; ** *p* < 0.01; *** *p* < 0.001), while the absence of an asterisk indicates no significant difference (*p* > 0.05).

As shown in Table 5, the addition of RP-Leu to the diet in late pregnancy had no significant effect on the carcass weight, mammary gland weight, and rumen weight of ewes (*p* > 0.05). The results of milk quality analysis (Figure 1A) showed that at day 30 postpartum, the contents of milk fat and total solids in the RP-Leu group were significantly higher than those in the control group (*p* < 0.05). In addition, as shown in Figure 1B, the concentrations of leucine metabolites KIC and HMB in the blood of the RP-Leu group were significantly increased at day 10 before parturition (*p* < 0.05), but there were no significant differences in the levels of blood antioxidant markers SOD, TOC, and MDA compared with the Con group (*p* > 0.05). Overall, perinatal RP-Leu supplementation improved milk composition and increased circulating leucine metabolites in ewes, but had no significant effects on maternal organ weights or antioxidant status.

### 3.3. Amino Acid-Based Targeted Metabolomics Analysis of Maternal Sheep Blood in Late Gestation

**Figure 2:**
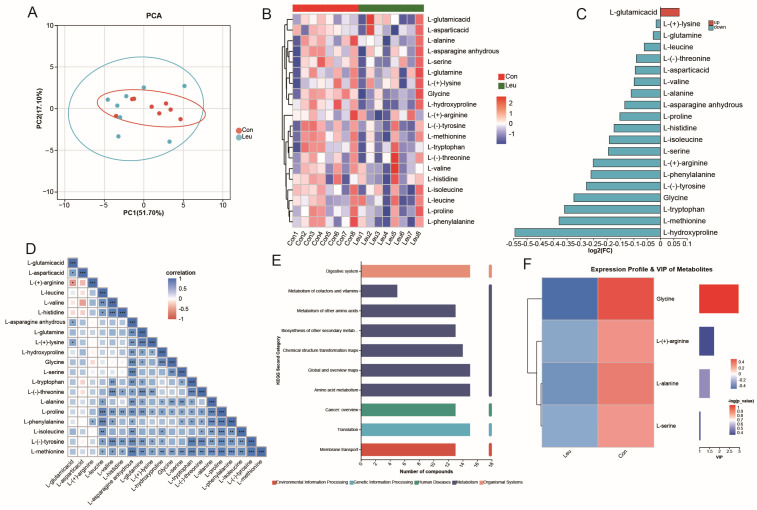
The effect of adding RP-Leu to ewes in late gestation on blood amino acid metabolism. (**A**) PCA score plot. Orange dots represent the Con group, and cyan dots represent the Leu group. Ellipses indicate the 95% confidence intervals for each group. (**B**) Hierarchical clustering heatmap. Rows represent individual amino acid metabolites, and columns represent samples. The color scale ranges from blue (low abundance) to red (high abundance) based on Z-score normalized values. (**C**) Volcano plot. Red columns indicate significantly upregulated metabolites, whereas blue columns represent metabolites showing a downward trend. (**D**) Pearson correlation heatmap. Displays the correlation coefficients among selected amino acids. Colors range from red (negative correlation) to blue (positive correlation). (**E**) KEGG pathway enrichment bubble/bar plot. The x-axis represents the number of differential metabolites enriched in each pathway, while different colors indicate distinct biological pathway categories. (**F**) Summary of differential metabolites based on VIP values and expression patterns. Note: Con = The control group; Leu = The leucine group. The data were obtained from slaughtered ewes, with 8 ewes in each group. Asterisks (*) above the error bar indicate significant differences between the two groups (* *p* < 0.05; ** *p* < 0.01; *** *p* < 0.001), while the absence of an asterisk indicates no significant difference (*p* > 0.05).

Principal component analysis (PCA) revealed a clear separation between the control (Con) and RP-Leu groups along principal component 1 (51.70%) and principal component 2 (17.10%) (Figure 2A). Partial overlap between the two groups was observed, with several samples from the Con group clustering within the RP-Leu group.

Among the detected metabolites, L-glutamic acid was significantly upregulated in the RP-Leu group, while several amino acids, including L-lysine, L-leucine, and L-isoleucine, showed differential abundance between groups (Figure 2B). Volcano plot analysis further identified L-glutamic acid as the most significantly upregulated metabolite with the highest fold change, whereas L-methionine was markedly downregulated (Figure 2C).

Correlation analysis demonstrated that L-glutamic acid was positively correlated with L-aspartic acid, L-arginine, and L-proline, whereas weak or negative correlations were observed with several branched-chain and aromatic amino acids (Figure 2D).

Pathway enrichment analysis indicated that differential metabolites were mainly involved in amino acid metabolism, biosynthesis of other amino acids, cofactor and vitamin metabolism, and membrane transport pathways. Pathways related to membrane transport and digestive system processes showed relatively higher enrichment significance (Figure 2E).

Based on the combined ranking of variable importance in projection (VIP) scores and log_2_ fold change, glycine, L-arginine, L-alanine, and L-serine were identified as key metabolites contributing to group separation, with glycine showing the most pronounced upregulation in the RP-Leu group (Figure 2F).

### 3.4. Effects of RP-Leu Supplementation on the Growth Performance of Offspring in Perinatal Ewes

**Figure 3:**
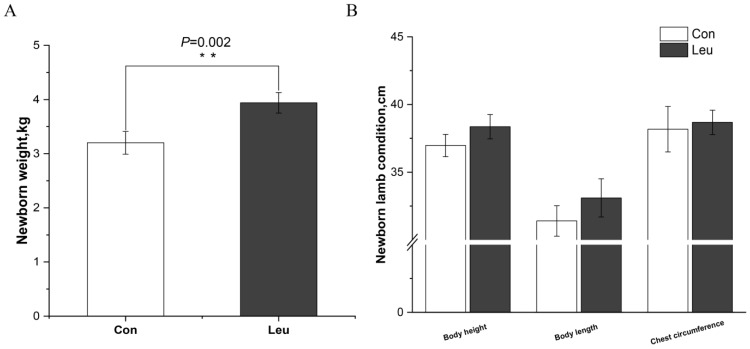
The effect of RP-Leu supplement in the late gestation on the weight (**A**), and body height, body length, and chest circumference (**B**) of newborn lambs. Note: Con = The control group; Leu = The leucine group. The data were obtained from the newborn lambs of non-slaughtered ewes, with 22 lambs in each group. Asterisks (*) above the error bar indicate significant differences between the two groups (** *p* < 0.01), while the absence of an asterisk indicates no significant difference (*p* > 0.05).

As shown in Table 6, compared with the Con group, the liver weight of fetal sheep (10 d before parturition) in the RP-Leu supplementary feeding group during late pregnancy increased significantly (*p* < 0.05), while there were no significant differences in fetal body weight, crown–rump length, and the weights of important organs such as the heart, spleen, lung, and kidney (*p* > 0.05). Meanwhile, there were also no significant changes in the levels of SOD and T-AOC in fetal blood (*p* > 0.05). In addition, as shown in Figure 3, the body weight of newborn lambs in the Leu group was significantly higher than that in the Con group (*p* < 0.05). Although there were no significant differences in body height, body length, and chest circumference between the two groups (*p* > 0.05). In conclusion, supplementary feeding of RP-Leu during pregnancy can promote the liver development of fetal sheep and significantly increase the body weight of newborn lambs, having a positive impact on the growth and development of offspring.

### 3.5. Perinatal Ewes Were Supplemented with RP-Leu to Promote Placental Development

**Figure 4:**
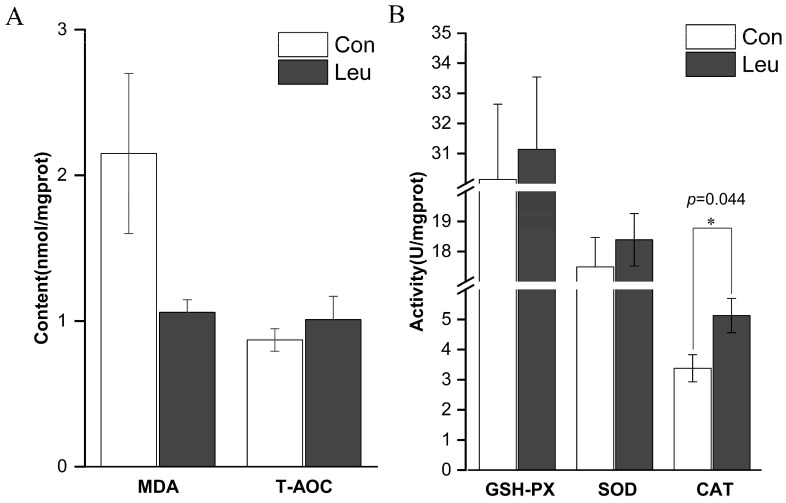
Effect of RP-Leu supplement on placental oxidative indices. (**A**) MAD and T-AOC concentrations. (**B**) GSH-PX, SOD, and CAT activity. Note: Con = The control group; Leu = The leucine group. The data were obtained from slaughtered ewes, with 8 ewes in each group. Asterisks (*) above the error bar indicate significant differences between the two groups (* *p* < 0.05), while the absence of an asterisk indicates no significant difference (*p* > 0.05).

Table 7 shows that compared with the control group, RP-Leu supplementary feeding significantly increased placental cotyledon density (*p* < 0.05), but had no significant effect on placental mass, total number of cotyledons, average diameter of cotyledons, total area of cotyledons, placental efficiency, and cotyledonary carrying efficiency (*p* > 0.05). In addition, Figure 4 shows that the concentration of CAT in the placenta of the Leu group increased significantly (*p* < 0.05), and the concentration of T-AOC, T-SOD, and GSH-PX increased, while MDA showed a downward trend, but the difference was not significant (*p* > 0.05).

**Figure 5:**
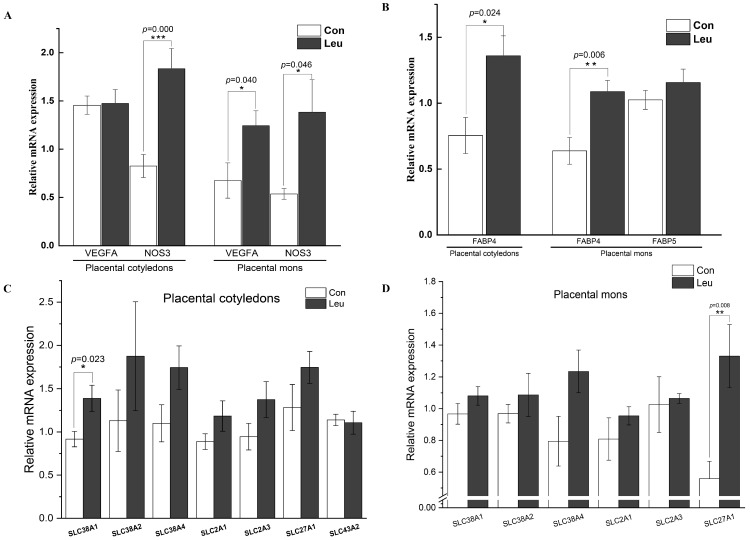
Effect of RP-Leu supplement on the transport of nutrients in the placenta of ewes in late gestation. (**A**) Expression of angiogenesis-related genes in cotyledon and caruncular tissues. (**B**) Expression of genes for fatty acid transporters in cotyledon and caruncular tissues. (**C**) Expression of amino acid transporter genes in cotyledon tissue. (**D**) Expression of amino acid transporter genes in caruncular tissue. Note: Con = The control group; Leu = The leucine group. The data were obtained from slaughtered ewes, with 8 ewes in each group. Asterisks (*) above the error bar indicate significant differences between the two groups (* *p* < 0.05; ** *p* < 0.01; *** *p* < 0.001), while the absence of an asterisk indicates no significant difference (*p* > 0.05).

The genes related to placental nutrient transport were determined, and the results showed that the relative expression levels of *NOS3*, *FABP4*, and *SLC38A1* in the placental cotyledons of the Leu group were significantly upregulated (*p* < 0.05), while there were no significant differences in the relative expression levels of *VEGFA*, *SLC38A4*, *SLC27A1*, *SLC2A1*, *SLC2A3*, and *SLC38A2* (Figure 5A–C). The relative expression levels of *VEGFA*, *NOS3*, *SLC27A1* and *FABP4* in the placenta and uterine carines of the Leu group were significantly upregulated (*p* < 0.05). There was no significant difference in the relative expression levels of *SLC38A1*, *SLC38A4*, *SLC2A1*, *SLC2A3*, *SLC38A2* and *FABP5* (Figure 5A,B,D).

### 3.6. Effects of Supplementation with RP-Leu on Placental Transcriptome Characteristics in Perinatal Ewes

**Figure A1:**
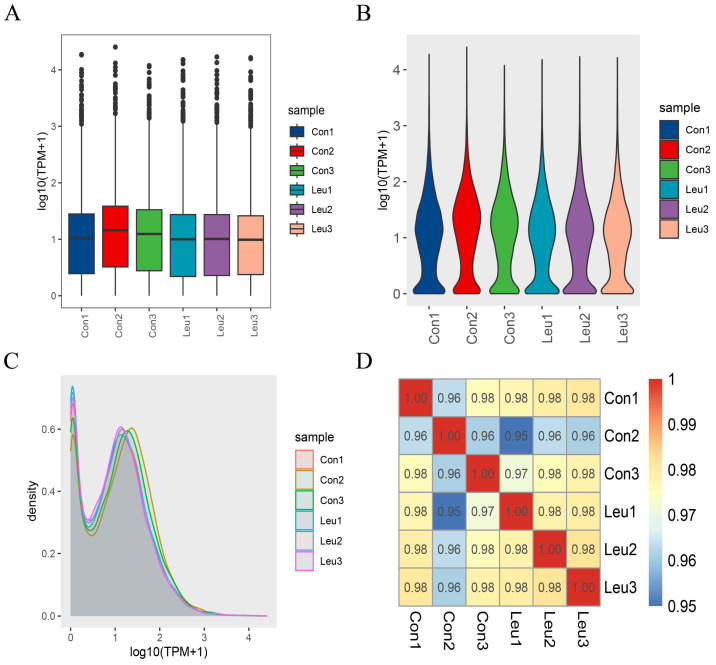
Placental mRNA expression characteristics of ewes. (**A**) Gene expression levels in each sample. (**B**) Violin plot of gene expression in each sample. (**C**) Distribution of gene expression density in the sample. (**D**) Pearson-correlated heat maps between different samples.

To validate the reliability of our RNA-seq datasets, we first examined the sequencing quality and alignment efficiency. The clean read counts, Q30 scores, and GC contents for each sample are detailed in Table A1, while the mapping rates and uniquely aligned reads are summarized in Table A2. Both tables indicated that the sequencing depth and quality were sufficient for downstream differential expression analysis. Subsequently, we assessed the overall expression landscape of the ovine placental transcriptome(Figure A1).The results showed that the intra-group repeatability was good, the inter-group differences were large, and the sequencing results were reliable.

**Figure 6:**
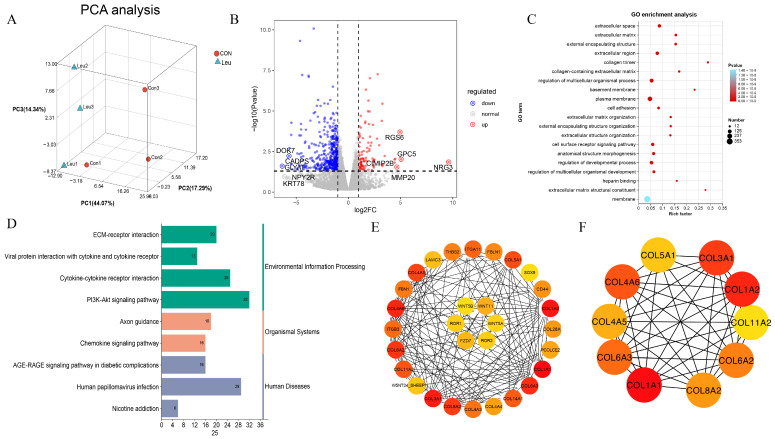
Placental mRNA expression enrichment analysis of ewes. (**A**) PCA diagram between sample relationships. (**B**) Volcano maps of the top 10 differential genes marked. (**C**) GO functional enrichment analysis of differential genes. (**D**) KEGG pathway enrichment analysis of differential genes. (**E**) Protein interaction network of the top 30 differentially expressed genes. (**F**) Protein interaction networks of the top 10 interconnected genes. Note: Con = The control group; Leu = The leucine group. The data were obtained from slaughtered ewes, with 3 ewes in each group.

Transcriptome sequencing of placental tissues identified 739 DEGs between the Leu and Con groups, and the results are shown in a volcano plot (Figure 6B). Of these DEGs, 515 were downregulated and 224 upregulated relative to the control. To clarify the associated biological functions and pathways, we carried out KEGG and GO enrichment analyses. GO enrichment revealed that the DEGs were predominantly associated with the extracellular region, collagen-containing extracellular matrix and basement membrane; with processes such as regulation of multicellular organismal processes, cell adhesion and extracellular matrix organization; and with molecular functions including receptor–ligand activity, transmembrane transporter activity and regulation of signal transduction receptors (Figure 6C).

KEGG pathway analysis revealed significant enrichment in pathways including ECM–receptor interaction, cytokine–cytokine receptor interaction, the PI3K-Akt signaling pathway, axon guidance, the chemokine signaling pathway, the AGE-RAGE signaling pathway in diabetic complications, and human papillomavirus infection (Figure 6D). Furthermore, a protein–protein interaction (PPI) network was constructed for the top 30 hub proteins (Figure 6E). Using the MCODE plugin in Cytoscape(version3.10.4), we identified the most significant cluster containing 10 highly interconnected proteins (Figure 6F), predominantly composed of collagen family members such as *COL5A1*, *COL4A6*, *COL4A5*, *COL6A3*, *COL1A1*, *COL8A2*, *COL6A2*, *COL11A2*, *COL1A2*, and *COL3A1*. Collectively, these results suggest that leucine may play a critical role in regulating extracellular matrix composition, cellular development, signal transduction, and transmembrane transport in the placenta.

### 3.7. Effects of Dietary Supplementation with RP-Leu During Pregnancy on the PI3K/Akt Signaling Pathway in Placental Cotyledon Tissue

**Figure 7:**
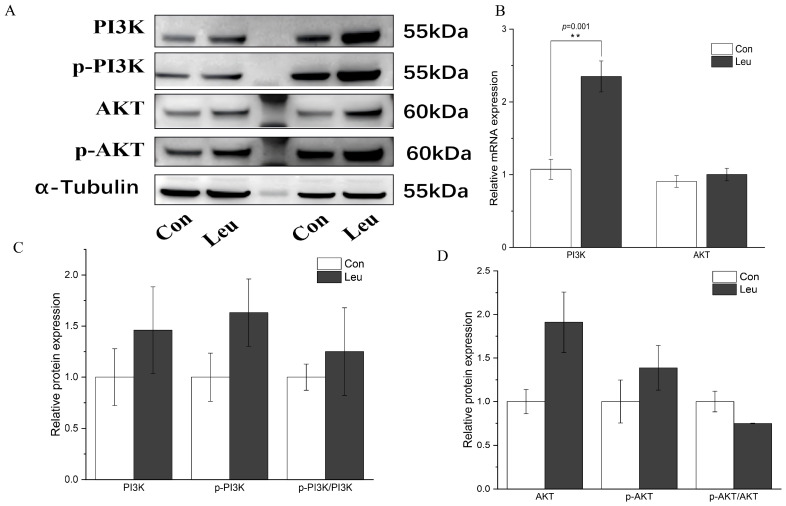
The effect of RP-Leu supplementation during pregnancy on the PI3K/Akt pathway in placental cotyledon tissue. (**A**) After supplementary feeding of RP-Leu in pregnant ewes, placental cotyletin proteins were detected using antibodies against PI3K, p-PI3K, AKT, p-Akt and a-tubulin. (**B**) Relative expression of PI3K and AKT genes in the placenta. (**C**,**D**) Density measurement values of the target protein. Note: Con = The control group; Leu = The leucine group. The data were obtained from slaughtered ewes, with 3 ewes in each group. Asterisks (*) above the error bar indicate significant differences between the two groups (** *p* < 0.01), while the absence of an asterisk indicates no significant difference (*p* > 0.05).

To investigate the potential relationship between leucine supplementation and the PI3K/Akt signaling pathway, the protein expression levels of PI3K, Akt, and their phosphorylated forms were examined in placental cotyledon tissues from the RP-Leu and Con groups. Western blot results showed that compared with the Con group, there were no significant changes in the protein expression levels of PI3K, p-PI3K, p-PI3K/PI3K ratio, Akt, p-Akt, and p-Akt/Akt ratio in the placental cotyledons of the Leu group (Figure 7A,C,D). However, fluorescence quantitative PCR detection showed that the relative mRNA expression level of PI3K in the placental cotyledons of the Leu group was significantly higher than that of the Con group (*p* < 0.05), while there was no significant difference in the mRNA expression level of Akt (Figure 7B).

Overall, RP-Leu supplementation during pregnancy upregulated PI3K transcription in placental cotyledon tissues, but had no significant effects on PI3K/Akt protein abundance or phosphorylation status, suggesting that leucine may modulate the PI3K/Akt pathway primarily at the transcriptional level rather than through activation of downstream proteins.

## 4. Discussion

The final two months of gestation represent a critical window for rapid fetal growth and tissue differentiation in sheep. Accordingly, the experimental period was defined from day 80 to day 140 of gestation, during which ewes were maintained on a consistent nutritional plane. Maternal nutrition during this stage plays a pivotal role in shaping endocrine function and metabolic status, thereby influencing fetal development [30,31]. Amino acids are not only fundamental substrates for protein synthesis but also serve as energy sources, metabolic intermediates, and key signaling molecules in cellular processes. During early gestation, amino acid requirements are relatively low and can generally be met by maternal reserves. However, in late gestation, the rapid acceleration of fetal growth markedly increases the demand for amino acids, making precise nutritional regulation essential. In addition to their fundamental role in protein synthesis, amino acids contribute to the formation and quality of colostrum and milk, which are critical determinants of neonatal survival and early postnatal growth [32]. Therefore, optimizing amino acid supply during late gestation is of particular importance for improving both maternal performance and offspring developmental outcomes.

In the present study, supplementation with RP-Leu significantly increased circulating levels of its key metabolic intermediates, KIC and HMB, indicating effective systemic availability of leucine. These metabolites are not only products of leucine catabolism but also functionally active compounds involved in energy metabolism and cellular signaling [5]. Consistent with this, milk fat and total solids content were significantly increased, in agreement with previous studies [33]. As milk fat represents the primary energy component of milk and a major contributor to total solids [34], its increase reflects an improvement in milk nutritional quality. It should be noted that individual milk yield was not measured in this study. Therefore, the above increases in milk fat and total solid contents only indicate an “increase in the percentage of components”, and it is still impossible to distinguish whether it is due to enhanced mammary gland synthesis or a concentration effect caused by a decrease in milk yield. Thus, the conclusion regarding the improvement of milk fat and total solids by RP-Leu should be cautiously limited to the level of “percentage increase”. In future research, it is necessary to conduct a comprehensive assessment in combination with milk yield data (such as daily milking volume or energy-corrected milk) to clarify the real impact mechanism of leucine on the nutritional quality of milk. The promotive effect of leucine on milk composition may be explained by multiple mechanisms. First, as an essential amino acid, leucine supports protein synthesis in mammary tissue by enhancing translational efficiency and ensuring adequate expression of genes involved in milk production [35]. Second, leucine and its metabolites act as signaling molecules that activate key metabolic pathways, including AMPK/mTOR and PPARγ, thereby regulating lipid synthesis, nutrient uptake, and energy utilization in mammary cells [36,37,38]. These combined effects likely contribute to the observed improvement in milk quality.

Targeted metabolomic analysis further demonstrated that leucine supplementation substantially reshaped the maternal metabolic profile. The clear separation between groups in PCA indicates a systemic metabolic shift induced by RP-Leu. The level of L-glutamic acid significantly increased, suggesting an increase in amino acid metabolic flux, which might be related to the substrate supply of the tricarboxylic acid cycle or the transamination process [39]. However, the specific mechanism and metabolic adaptive significance need to be further verified in combination with ketone bodies, respiratory quotient or other energy markers. The positive correlations between glutamic acid and amino acids such as arginine, aspartic acid, and proline further suggest activation of the urea cycle–nitric oxide (NO) synthesis pathway [40,41,42,43]. This pathway plays a critical role in regulating uterine and placental blood flow, thereby facilitating nutrient delivery to the fetus [42,43]. Interestingly, despite dietary leucine supplementation, plasma levels of leucine and isoleucine remained relatively stable, while methionine showed a decreasing trend. This observation highlights the tight homeostatic regulation of branched-chain amino acid metabolism in pregnant ewes [44]. The reduction in methionine may reflect increased utilization of methyl donors for fetal growth and epigenetic modifications [45]. In addition, key metabolites identified by VIP analysis, including glycine, arginine, alanine, and serine, indicate enhanced one-carbon metabolism, antioxidant capacity, and vascular adaptation. For example, glycine contributes to nucleotide synthesis and glutathione production, supporting both fetal development and maternal antioxidant defense [46,47], while arginine plays a central role in NO-mediated vasodilation [48].

Consistent with these metabolic adaptations, RP-Leu supplementation significantly increased lamb birth weight and fetal liver weight, indicating enhanced fetal growth. Birth weight is a critical determinant of neonatal viability, survival rate, and subsequent growth performance [49,50,51,52,53,54]. Although the circulating leucine levels in this study did not increase significantly due to RP-Leu supplementation, the key metabolic intermediates KIC and HMB both increased significantly. Therefore, the observed growth-promoting effect is likely not directly mediated by leucine itself, but rather exerts its influence through its metabolites. KIC and HMB have been reported to independently activate the mTOR signaling pathway, promote protein synthesis and inhibit protein degradation [55,56]; meanwhile, changes in leucine metabolites may trigger systemic metabolic reprogramming, such as enhancing the utilization efficiency of maternal nutrients or altering the placental transport capacity [57]. In conclusion, supplementing RP-Leu may indirectly promote fetal growth by increasing the circulating levels of active metabolites such as KIC and HMB, rather than relying on the increase in leucine concentration itself. The observed increases in fetal organ weights, particularly in metabolically active organs such as the liver, further support enhanced developmental potential. The fetal liver is a central organ for metabolism, nutrient storage, and endocrine regulation, and its development is closely linked to overall fetal health [58]. Leucine may promote liver development through multiple pathways, including stimulation of hepatocyte proliferation, enhancement of protein synthesis via mTOR activation, improvement of mitochondrial energy metabolism, and modulation of maternal hormonal signals such as insulin and growth hormone [59,60,61,62,63,64].

Placental adaptations appear to represent a central mechanism underlying these effects. The placenta is the primary interface for nutrient exchange between mother and fetus, and its structure and function directly determine fetal growth potential. In this study, RP-Leu supplementation significantly increased placental cotyledon density, suggesting an expansion of the maternal–fetal exchange surface. This effect is particularly important in prolific breeds such as Hu sheep, where improved placental efficiency can alleviate intrauterine competition among fetuses. The increase in placental catalase (CAT) activity indicates an enhanced ability to scavenge hydrogen peroxide. Given that the placenta has high metabolic activity and is vulnerable to oxidative stress, improving local antioxidant defense is crucial for maintaining placental integrity and function [65]. Meanwhile, in this study, there were no significant changes in maternal plasma antioxidant markers (SOD, TOC, MDA), which is in contrast to the elevated placental CAT activity. This difference suggests that the supplementation of RP-Leu may primarily induce a local antioxidant adaptive response in the placenta rather than cause changes in the systemic redox state. As an organ with high oxygen consumption and metabolic activity, the placental antioxidant enzyme system may be more sensitive to the signals of leucine or its metabolites (such as KIC, HMB), while plasma markers reflect the overall systemic level and may be buffered and balanced by the metabolism of multiple tissues. Therefore, the observed upregulation of placental CAT activity may represent a tissue-specific protective mechanism aimed at enhancing the placenta’s own ability to resist oxidative damage rather than achieving this by improving the systemic antioxidant capacity.

At the molecular level, leucine supplementation upregulated key genes involved in vascular function and nutrient transport, including *NOS3*, *VEGFA*, *SLC38A1*, *SLC27A1*, and *FABP4.* However, there were differences in their expression profiles between the cotyledons (fetal side) and the maternal caruncles (maternal side): SLC38A1 and FABP4 were upregulated in the cotyledons, while *SLC27A1* and *FABP4* were upregulated in the maternal caruncles. This regional difference may reflect the different requirements and transport strategies for nutrients on the two sides of the placenta, specifically, the upregulation of *SLC38A1* (system A amino acid transporter) in the cotyledon. Although the circulating leucine itself is not elevated, *SLC38A1* can transport multiple small-molecule neutral amino acids including leucine. The increased expression of *SLC38A1* may enhance the uptake of leucine metabolites (such as KIC, HMB) or other functional amino acids (such as glutamine, alanine) on the fetal side, thus supporting fetal protein synthesis and growth [5]. Meanwhile, the upregulation of FABP4 in the cotyledon facilitates the intracellular transport and metabolism of fatty acids [66]. On the other hand, the upregulation of *SLC27A1* (long-chain fatty acid transporter) and *FABP4* in the maternal caruncle may enhance the transmembrane transport and utilization of fatty acids from maternal blood to the basal side of the placenta, providing lipid substrates for the placental energy metabolism and transport function. These coordinated but region-specific gene upregulations jointly improve placental angiogenesis (*VEGFA*), vasodilation (*NOS3*) [67], and the nutrient transport capacity on both sides [68], ultimately increasing placental efficiency and promoting fetal growth. Transcriptomic analysis further supported these findings, with differentially expressed genes significantly enriched in pathways related to extracellular matrix (ECM) organization, cell adhesion, and signaling pathways such as PI3K-Akt. ECM remodeling is essential for maintaining placental structure and facilitating cell migration, differentiation, and nutrient exchange [69,70]. Transcriptome data show that the ECM–receptor pathway and collagen gene expression are upregulated, suggesting that the ECM components of the placenta have undergone remodeling. However, its specific impact on the structure and function of the placenta (such as supporting an increase in villus density, tissue fibrosis or repair response) still needs to be directly verified through histological staining (such as Masson tricolor staining to observe collagen deposition and distribution). The current data can only reveal the changing trends at the molecular level. The functional and structural conclusions should be regarded as speculative hypotheses. In addition, activation of PI3K-Akt and cytokine-related pathways suggests enhanced regulation of cell proliferation, survival, and metabolic adaptation, further contributing to improved placental function.

Preliminary cost–benefit analysis indicates that in commercial sheep farms, supplementing each pregnant ewe with 19 g/d of RP-Leu (containing approximately 10 g of effective leucine) costs about 43.4 CNY per ewe over the 70 d experimental period (from 80 d to 150 d of gestation), based on a raw leucine price of 62 CNY/kg (total cost of 1302 CNY for 30 ewes). Lambs in the Leu group had a 0.74 kg higher birth weight than those in the Con group. At a market price of 30 CNY/kg for lambs, the 60 lambs generated an additional revenue of approximately 1332 CNY, resulting in a net profit of about 30 CNY, which essentially achieves break-even. Considering the indirect benefits of increased birth weight, such as a shortened fattening period and improved survival rate, this strategy may yield a modest profit, indicating preliminary feasibility and potential for practical application.

Taken together, these results indicate that leucine indirectly regulates placental function through multiple coordinated mechanisms, including structural remodeling, promotion of angiogenesis and blood perfusion, and upregulation of nutrient transport systems. These integrated effects ultimately improve maternal–fetal nutrient exchange and support optimal fetal growth and development.

The following limitations of this study need to be taken into account when interpreting the conclusion: only a single dose of leucine (19 g/d per sheep) was tested, and no multi-dose gradient was set, making it impossible to determine the dose-effect relationship and the optimal level; some indicators were not measured completely, including the failure to determine the milk production of ewes (only the concentration data of milk components) and the lack of functional markers for liver weight gain in lambs (unable to distinguish between hyperplasia/hypertrophy and metabolite accumulation). In this study, direct functional transport assays (such as the net trans-placental substrate transport rate), placental hemodynamic assessments, or histomorphometric analyses were not conducted. Therefore, the proposed “improvement in placental nutrient transport capacity” based on the upregulation of transporter gene expression remains an indirect inference. Future studies should combine placental perfusion techniques, microvascular imaging, and stereological quantitative methods to more reliably verify the effects of leucine on placental function. The tracking period was only until the lambs were 30 d old, and the effects of leucine on the subsequent reproductive performance of ewes and the long-term growth of lambs were not evaluated. In addition, the research results are based on the Hu sheep and the single-pen refined and coarse feed separation model. Caution should be exercised when extending them to other breeds or rearing conditions. In conclusion, this study is a preliminary exploratory finding. The above conclusions need to be further verified in future multi-dose designs, more comprehensive index measurements, and long-term follow-up studies.

## 5. Conclusions

There is a positive correlation between the supplementation of RP-Leu in the late pregnancy and the improvement of growth performance in Hu sheep offspring. Transcriptomic and metabolomic data suggest that this association may involve the adjustment of amino acid metabolic profiles, changes in placental structure development, and the upregulation of the expression of genes related to nutrient transport. Therefore, RP-Leu may be associated with the improvement of offspring growth performance through indirect pathways (such as regulating maternal amino acid metabolism and affecting placental development). However, the above mechanisms still need to be further verified through functional experiments such as nutrient transport rate measurement and placental perfusion studies. Overall, RP-Leu supplementation may improve maternal–fetal nutrient exchange efficiency and represents a practical nutritional strategy for enhancing reproductive performance and offspring development in sheep.

## Figures and Tables

**Table 1:** Composition and nutrient levels of experimental diets (DM basis, %).

Concentrate Supplementary Materials			Roughage		
Items	Con	Leu	Items	Con	Leu
Corn	33.82	33.82	Whole Corn Silage	32.25	32.25
Soybean Meal	11.84	11.84	Rice Straw	27.65	27.65
Corn Bran	9.47	9.47	Soybean Residue	27.65	27.65
Soybean Hulls	11.64	11.64	Peanut Vine	5.53	5.53
Rice Bran	7.89	7.89	Soybean Straw Powder	4.15	4.15
Rice Mill By-Product	7.06	7.06	White Wine Lees	2.77	2.77
Textured Soy Protein	5.91	5.91	Total	100.00	100.00
Waste Milk Powder	3.95	3.95			
Wheat Bran	4.93	4.93			
Sodium Bicarbonate	0.59	0.59			
Sodium Chloride	0.46	0.46			
Premix	2.45	2.45			
Total	100.00	100.00			
Chemical Composition		Con		Leu	
Dry Matter DM, % DM		59.90		59.90	
Crude Protein CP		12.63		12.63	
Crude Fat EE, % DM		4.29		4.29	
Neutral Detergent Fiber NDF, % DM		34.73		34.73	
Acid Washing Fiber ADF, % DM		14.81		14.81	

Note: Con = The control group; Leu =The leucine group. Premix is the amount provided per kilogram of feed: Vitamin A ≥ 10 KIU, Vitamin D3 ≥ 1.5 KIU, Vitamin E ≥ 125 IU, Manganese ≥ 0.5 g, Iron ≥ 0.6 g, Zinc ≥ 0.5 g, Copper ≥ 0.1 g, Calcium: 7–14.5%, Phosphorus ≥ 1.5%, Moisture ≤ 12%.

**Table 2:** Primer sequences and amplicon information.

Gene	Primer Sequence (5′-3′)	Product Size (bp)	Accession Number
*GAPDH*	F-ATCAAGTGGGGTGATGCTGG	120	NM_001357943.2
	R-GGTTCACGCCCATCACAAAC		
*VEGFA*	F-GGCTGCTGTAATGACGAA	104	NM_001025110.1
	R-TCTCCTATGTGCTGGCTT		
*NOS3*	F-AGGCATCACCAGGAAGAA	118	NM_001129901.1
	R-CTCAGAGGCGTACAGGAT		
*SLC38A1*	F-CAACCTCCTTGGGCATGTCT	96	XM_027967425.3
	R-TGTGGTGCCAAAGACCTGTT		
*FABP4*	F-AGGTGCTCTGGTACAAGT	117	NM_001114667.1
	R-ACTCTGGTAGCAGTGACA		
*FABP5*	F-CTGTCTGCAACTTTACGGAT	119	NM_001145180.1
	R-ATTGTTCATGACGCATACCAC		
*SLC38A4*	F-CAAGGGCTCTCTTCATGGCA	103	XM_042246822.2
	R-TTTGCATCTTCCTCCGGGAC		
*SLC27A1*	F-CTTACCGGACACCCAACTCC	98	XM_042249696.1
	R-AGGAGTAGTGCCCAAATGCC		
*SLC2A1*	F-TGGGAAAGTCCTTTGAGATGC	107	XM_027968628.3
	R-GGTCAGGCCGCAGTACACA		
*SLC2A3*	F-GTCACAGTGCTGGAGCTCTT	115	NM_001009770.1
	R-CACCGATAGTGGCGTAGACC		
*SLC38A2*	F-TGGGAAGCTCACAGCATCTG	110	XM_004006421.5
	R-GGCCACTGGTGTATCCCAAA		

**Table 3:** Relevant information about the antibodies used.

Antibodies	Cat No.	Source	Dilution of WB
Anti-α-Tubblin Antibody	80762-1-RR	Proteintech (Rosemont, IL, USA)	1:10,000
Anti-PI3K Antibody	MCA2673GA	Bio-Rad (Hercules, CA, USA)	1:1000
Anti-phospho-PI3K Antibody	AF3423	Affinity (Cincinnati, OH, USA)	1:2000
Anti-Akt Antibody	DF7588	Affinity (Cincinnati, OH, USA)	1:1000
Anti-phospho- Akt Antibody	DF7587	Affinity (Cincinnati, OH, USA)	1:1000
Goatanti-Rabbit IgG	AS014	Abclonal(Woburn, MA, USA)	1:8000

**Table 4:** Determination of leucine through rumen rate at different time periods.

Time	Amino Acid Release Rate %	Amino Acid Passage Rate %
6 h	2.31	97.69
12 h	4.10	95.90
24 h	5.33	94.67
48 h	7.73	92.27
RL-Leu content		54.87

**Table 5:** Determination of partial slaughter performance and serum biomarkers in ewes during late pregnancy.

Item	Con	Leu	*p*-Value
Ewe carcass weight (kg)	22.50 ± 0.51	23.20 ± 0.49	0.38
Breast weight (g)	873.43 ± 58.91	1009.53 ± 43.19	0.14
Rumen dry weight (g)	599.40 ± 16.67	625.43 ± 16.50	0.33
Rumen wet weight (kg)	2.74 ± 0.22	3.27 ± 0.15	0.12
SOD (U/mgprot)	965.74 ± 1.48	850.54 ± 4.72	0.67
TOC (nmol/mgprot)	0.50 ± 0.0088	0.46 ± 0.0058	0.44
MDA (nmol/mgprot)	3.01 ± 0.13	2.79 ± 0.091	0.18

Note: Con = The control group; Leu = The leucine group. The data were obtained from slaughtered ewes, with 8 ewes in each group.

**Table 6:** Effects of RP-Leu supplementation in ewes in the second and third trimesters of pregnancy on fetal body weight, organs and antioxidant indices.

Item	Con	Leu	*p*-Value
Fetal sheep weight/(kg)	2.39 ± 0.13	2.41 ± 0.17	0.95
Head and hip length/(cm)	39.67 ± 0.92	41.00 ± 0.52	0.24
Cardiac weight/(g)	15.82 ± 1.33	17.66 ± 1.39	0.37
Liver weight/(g)	52.60 ± 0.99	68.24 ± 1.01 *	0.01
Spleen weight/(g)	4.08 ± 0.15	4.13 ± 0.12	0.82
Lung weight/(g)	58.20 ± 3.33	51.70 ± 2.86	0.17
Kidney weight/(g)	20.13 ± 0.57	22.95 ± 1.18	0.06
SOD (U/mgprot)	144.06 ± 3.62	207.41 ± 5.24	0.25
TOC (nmol/mgprot)	0.56 ± 0.0077	0.53 ± 0.016	0.45

Note: Con = The control group; Leu = The leucine group. The data were obtained from fetal sheep of slaughtered ewes, with 8 fetal sheep in each group. Asterisks (*) indicate significant differences between the two groups (* *p* < 0.05), while the absence of an asterisk indicates no significant difference (*p* > 0.05).

**Table 7:** Statistics of placental traits.

Item	Con	Leu	*p*-Value
Fetal sheep litter weight, kg	4.79 ± 0.41	4.81 ± 0.53	0.97
The average newborn weight of fetal sheep, kg	2.39 ± 0.13	2.41 ± 0.17	0.95
Placental mass, g	1,695.73 ± 31.54	1,660 ± 5.77	0.33
Total number of cotyledon leaves, pieces	98.33 ± 2.85	100.67 ± 1.20	0.49
The average diameter of the cotyledon, cm	27.30 ± 1.63	24.05 ± 0.38	0.12
Total area of cotyledon, cm^2^	563.25 ± 3.96	557.53 ± 10.44	0.64
Cotyledon density, n/g^2^	0.047 ± 0.0033	0.070 ± 0.0056 *	0.024
Placental efficiency	2.80 ± 0.017	3.20 ± 0.32	0.34
Cotyledonary carrying efficiency, g/cm^2^	12.16 ± 0.16	11.23 ± 0.43	0.11

Note: Con = The control group; Leu = The leucine group. The data were obtained from slaughtered ewes, with 8 ewes in each group. Asterisks (*) indicate significant differences between the two groups (* *p* < 0.05), while the absence of an asterisk indicates no significant difference (*p* > 0.05).

## Data Availability

The raw sequencing data of the RNA-seq section have been submitted to the NCBI database, with the accession number PRJNA1475953.

## Appendix A

### Filtering and Quality Evaluation of Ewe Placental Transcriptome Sequencing Data

Table A1 shows that the pass rate of sequencing results in all three groups is higher than 90%, indicating that the reads obtained in this study are of good quality and can be used for further research.

**Table A1:** Quality control of transcriptome sequencing data.

Sample	Raw Reads	Raw Bases	Clean Reads	Clean Bases	Error Rate (%)	Q20 (%)	Q30 (%)	GC Content (%)
Con1	51,150,658	7,723,749,358	50,745,204	7,626,462,590	0.0120	98.77	96.05	50.56
Con2	53,274,514	8,044,451,614	52,995,782	7,966,890,915	0.0116	99.02	96.79	50.26
Con3	41,544,086	6,273,156,986	41,221,314	6,199,845,210	0.0121	98.69	95.76	52.57
Leu1	48,046,902	7,255,082,202	47,686,946	7,171,904,623	0.0119	98.84	96.25	49.58
Leu2	43,376,780	6,549,893,780	43,047,188	6,475,336,910	0.0119	98.82	96.18	50.17
Leu3	49,001,772	7,399,267,572	48,641,216	7,317,483,868	0.0119	98.80	96.13	50.39

Note: (1) Sample: Sample name. (2) Raw reads: The total number of entries in the original sequencing data (reads, representing sequencing reads, one read is one). (3) Raw bases: The total amount of raw sequencing data (i.e., the number of raw reads multiplied by the length of reads). (4) Clean reads: The total number of entries in the sequencing data after quality control. (5) Clean bases: The total amount of sequencing data after quality control (i.e., the number of clean reads multiplied by the length of reads). (6) Error rate (%): The average error rate of sequencing bases corresponding to quality control data is generally less than 0.1%. (7) Q20 (%), Q30 (%): The quality of the sequencing data after quality control; Q20 and Q30 refer to the percentage of bases with sequencing quality above 99% and 99.9% of the total bases; generally, Q20 is above 85%, and Q30 is above 80%. (8) GC content (%): The percentage of the sum of G and C bases corresponding to the quality control data to the total bases.

The results of sequence alignment analysis using HISAT2(Version 2.2.1) software showed that the matching rates of the three Con groups and the three Leu groups were greater than 90%, indicating that the selected reference gene combination was suitable and met the requirements of subsequent analysis.

**Table A2:** Statistics of comparison results.

Sample	Total Reads	Total Mapped	Multiple Mapped	Uniquely Mapped
Con1	50,745,204	49,531,939 (97.61%)	2,269,569 (4.47%)	47,262,370 (93.14%)
Con2	52,995,782	51,999,895 (98.12%)	2,298,004 (4.34%)	49,701,891 (93.78%)
Con3	41,221,314	40,249,662 (97.64%)	1,676,250 (4.07%)	38,573,412 (93.58%)
Leu1	47,686,946	46,441,414 (97.39%)	2,649,154 (5.56%)	43,792,260 (91.83%)
Leu2	43,047,188	41,998,835 (97.56%)	2,083,059 (4.84%)	39,915,776 (92.73%)
Leu3	48,641,216	47,423,562 (97.5%)	2,580,683 (5.31%)	44,842,879 (92.19%)

Note: (1) Sample: This is the name of the sample. (2) Total reads: The statistics of the number of sequences after filtering of sequencing sequences (i.e., Clean reads). (3) Total mapped: The number of Clean reads that can be located on the genome. (4) Multiple mapped: The number of Clean reads with a unique alignment position on the reference sequence. (5) Uniquely mapped: The number of Clean reads with a unique alignment position on the reference sequence.

The gene distribution violin diagram (Figure A1B) and heat map (Figure A1D) show that the reproducibility within the group was good, the difference between the groups was large, and the sequencing results were reliable.
